# Wavelet-prior-guided mamba network for accurate and efficient rice disease recognition

**DOI:** 10.3389/fpls.2026.1935930

**Published:** 2026-09-01

**Authors:** Wei Zhang, Tao Zhang, Shengyue Chen, Haoran Li, Chunhui Zhang, Shuai Wang

**Affiliations:** 1 College of Mechanical and Electrical Engineering Inner Mongolia Agricultural University, Hohhot, China; 2 School of Mechanical Engineering, Guiyang University, Guiyang, China

**Keywords:** frequency-domain feature modeling, mamba network, precision agriculture, rice disease recognition, wavelet prior

## Abstract

Rice disease recognition is of great importance for sustainable agricultural production. Ho wever, most existing methods mainly rely on spatial-domain feature modeling and insufficiently exploit frequency-domain information, making it difficult to fully characterize the texture and structural patterns of diseased images across different frequency components. In addition, conventional convolutional neural networks have limitations in modeling long-range dependencies, which may reduce their ability to recognize lesion regions with uneven distributions or highly variable appearances. To address these challenges, this study proposes a Wavelet-Prior-Guided Mamba network, termed WPMamba, for rice disease recognition. Specifically, discrete wavelet transform is introduced to decompose image features into different frequency components, thereby explicitly incorporating frequency-domain prior information. The decomposed features are further integrated with Mamba modules to enhance global feature modeling and representation capability. Moreover, a prior-guided selective fusion (PGSF) module is designed to adaptively fuse spatial-domain and frequency-domain features, while a lesion-aware spatial attention (LASA) module is introduced to guide the network toward disease-relevant regions. Experimental results on a rice disease dataset demonstrate that the proposed WPMamba achieves an accuracy of 95.72%, outperforming several mainstream models. Meanwhile, WPMamba contains only 1.21 M parameters and requires 0.49 GFLOPs, indicating its favorable balance between recognition performance and computational efficiency. These results suggest that WPMamba provides an effective and efficient solution for intelligent rice disease recognition.

## 1 Introduction

Rice is one of the most important staple crops worldwide, and its yield and quality are directly associated with food security and sustainable agricultural development (Pi et al., 2026; Lu et al., 2025). However, in practical production, rice is highly susceptible to various diseases, such as bacterial leaf blight, rice blast, and brown spot. These diseases not only impair leaf function but also affect plant development at different growth stages, leading to substantial yield losses and, in severe cases, complete crop failure (Liu et al., 2024). In addition, different rice diseases often exhibit similar symptoms at early stages, which increases the difficulty of accurate identification. Once disease outbreaks spread, they become difficult to control (Sharif et al., 2026). Therefore, rapid and accurate recognition of rice diseases is essential for timely disease management, reducing economic losses, and ensuring food security.

Traditional rice disease recognition mainly relies on the empirical judgment of agricultural experts or farmers, who typically classify diseases by observing changes in leaf color, lesion morphology, and spatial distribution patterns (Song et al., 2024). This approach can meet the needs of small-scale cultivation to some extent; however, its accuracy is highly dependent on individual experience and is inherently subjective, often leading to considerable variability among different observers (Wei et al., 2025; Zhang et al., 2025a). Moreover, manual recognition is time-consuming and labor-intensive, making efficient monitoring difficult in large-scale field environments (Zhang et al., 2022; Zeng and He, 2024). Some diseases also show inconspicuous symptoms at early stages or share similar visual characteristics with other diseases, further increasing the difficulty of manual diagnosis. Consequently, traditional experience-based recognition methods have clear limitations in terms of efficiency, accuracy, and robustness.

With the development of image processing techniques, researchers have attempted to use traditional machine learning methods for rice disease recognition. These methods generally involve several steps, including image preprocessing, handcrafted feature extraction, and classifier design. Commonly used features include color, texture, and shape descriptors (Mukherjee et al., 2025). The extracted handcrafted features are then combined with classifiers, such as support vector machines (SVM), k-nearest neighbors (KNN), or decision trees, to perform disease classification (Tiwari et al., 2025; Zhang et al., 2025b). Compared with manual diagnosis, these methods improve recognition efficiency and objectivity to some extent (Zhang et al., 2025). However, their performance is highly dependent on manually designed feature representations, which are often insufficient to characterize fine-grained texture variations and structural differences in complex disease images (Radočaj et al., 2026). In addition, under varying illumination conditions, background interference, or diverse disease morphologies, handcrafted features usually exhibit limited robustness, thereby restricting their practical applicability in complex agricultural scenarios.

In recent years, deep learning methods have achieved substantial progress in automatic rice disease recognition, with most mainstream approaches relying on convolutional neural networks (CNNs) for spatial-domain feature modeling (Ismail et al., 2026; Kang et al., 2025; Zhang et al., 2026; Zhang et al., 2025d). Wang et al. introduced an attention mechanism into the MobileNet framework and combined it with Bayesian optimization for hyperparameter tuning, achieving effective multi-class rice disease classification while maintaining a low parameter count (Wang et al., 2021). Subsequently, researchers further integrated attention modules with deeper backbone networks to enhance the discriminative capability of key features. Al-Gaashani et al. designed a kernel attention module based on ResNet50, which captures contextual dependencies within feature maps and guides the model to focus on lesion regions, achieving high recognition accuracy on multi-class rice disease datasets (Al-Gaashani et al., 2023). Jiang et al. combined DenseNet with the squeeze-and-excitation (SE) channel attention mechanism, using dense connections to enhance multi-scale feature reuse and achieving an accuracy of 99.4% in a five-class rice disease recognition task (Jiang et al., 2023). Nevertheless, the inherent limitation of local receptive fields in CNNs has become increasingly evident. CNN-based methods remain insufficient in modeling long-range spatial dependencies in diseased images, which limits their ability to recognize lesion regions with scattered distributions or highly variable appearances.

To compensate for the limited global modeling capability of CNNs, researchers have begun to introduce Transformer architectures. AL et al. combined a deep Vision Transformer with an optimization algorithm and used the multi-head self-attention mechanism to capture long-range dependencies among image patches, achieving promising performance in multi-class rice disease classification (Al et al., 2025). Hassan et al. proposed IEViT, which integrates multi-kernel convolution with Transformer modules to simultaneously extract local texture and global structural features, demonstrating strong generalization performance in multi-class plant disease recognition (Hassan et al., 2025). However, the computational complexity of Transformer self-attention increases quadratically with sequence length, resulting in large parameter sizes and high computational costs. This makes Transformer-based models difficult to deploy in resource-constrained agricultural scenarios. To address the efficiency bottleneck of Transformers, state space models (SSMs), represented by Mamba, have recently attracted increasing attention. Jiao et al. proposed VMamba, which introduces a two-dimensional selective scan module (SS2D) to transfer Mamba’s linear-complexity long-range modeling capability to vision tasks, showing better performance and computational efficiency than Transformers of comparable scale in image classification and other tasks (Jiao et al., 2024).

Despite these advances, existing spatial-domain methods have not explicitly modeled frequency-domain information. Related studies have shown that introducing discrete wavelet transform into deep neural networks can decompose features into different frequency sub-bands, thereby enriching feature representations and compensating for the insufficient fine-grained texture characterization of purely spatial-domain methods without increasing the number of parameters (Pi et al., 2026). However, how to effectively integrate frequency-domain priors with efficient global sequence modeling, and how to design adaptive spatial-frequency fusion and lesion-aware attention mechanisms for diseased images, remain insufficiently explored in the field of rice disease recognition.

To address the above issues, this study proposes a Wavelet-Prior-Guided Mamba network, termed WPMamba, to improve the accuracy and efficiency of rice disease recognition. By introducing discrete wavelet transform, the proposed method decomposes feature maps into different frequency components, thereby explicitly incorporating frequency-domain prior information. The decomposed features are further integrated with Mamba modules to achieve global feature modeling. Meanwhile, a dual-branch architecture is designed to collaboratively learn spatial-domain and frequency-domain features, and an attention mechanism is employed to further enhance the representation of key lesion regions.

The main contributions of this study are summarized as follows:

1. A wavelet-prior-guided feature modeling strategy is proposed to introduce frequency-domain information into rice disease recognition, enhancing the model’s ability to represent complex texture and structural characteristics at the feature level.
2. A prior-guided selective fusion (PGSF) module is designed to adaptively integrate spatial-domain and frequency-domain features. Combined with Mamba-based global modeling, this module improves the effectiveness and consistency of feature representations.
3. A lesion-aware spatial attention (LASA) module is developed to highlight key disease regions and suppress background interference, significantly improving recognition performance while maintaining low computational complexity.

## 2 Materials and methods

### 2.1 Data collection

**Figure 1:**
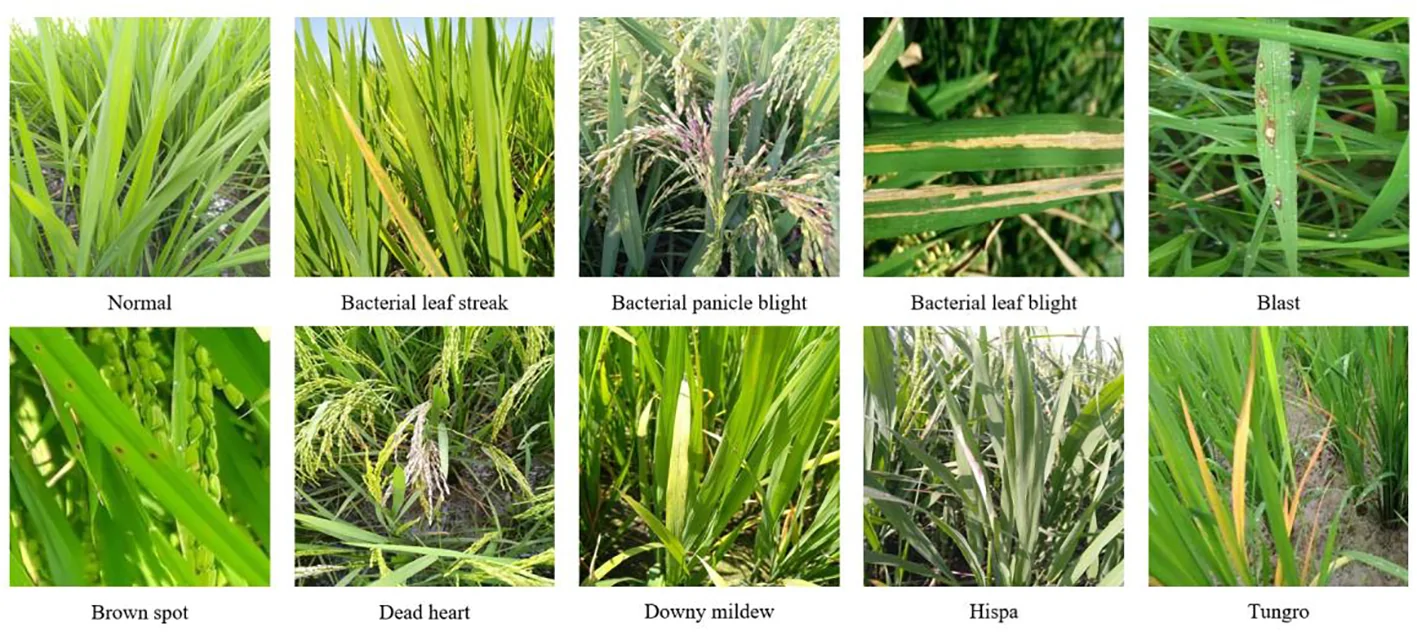
Representative images from the rice disease dataset.

**Figure 2:**
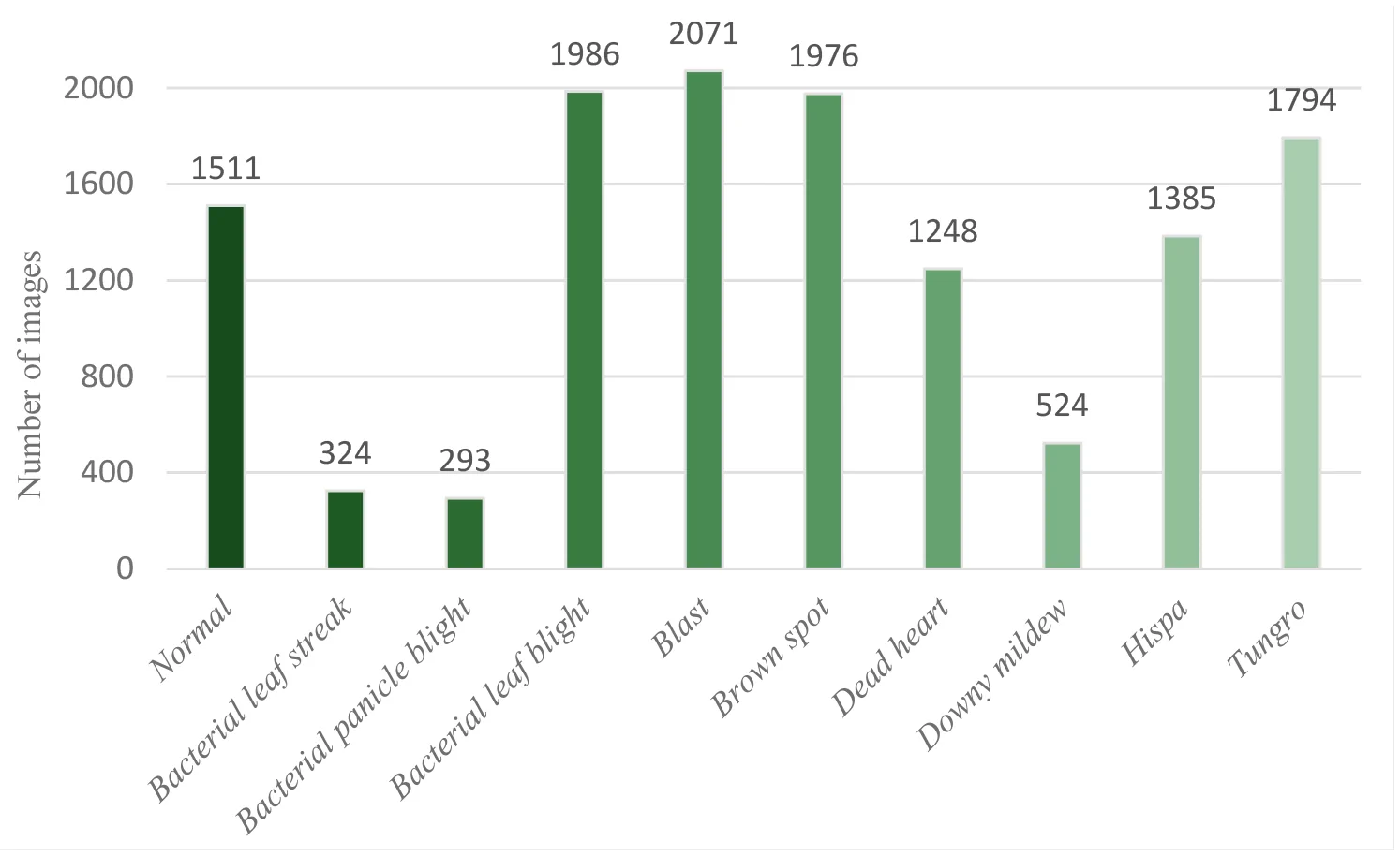
Statistical results of sample size for each rice disease species.

The rice disease images used in this study were mainly collected from public datasets and online web resources, such as *Kaggle*. The public dataset contains 5,932 images of diseased rice leaves (Sethy et al., 2020), covering four typical disease categories: bacterial leaf blight, blast, brown spot, and tungro. In addition, image data were further collected from the *Kaggle* platform and related agricultural disease image resource websites, resulting in 7,180 additional images. This process effectively expanded the sample size and improved the diversity of disease categories.The additionally collected data include nine rice disease categories, namely *bacterial leaf streak*, *bacterial panicle blight*, *bacterial leaf blight, blast, brown spot, dead heart, downy mildew, hispa, and tungro*, as well as images of healthy rice leaves, as shown in **Figure 1**. Finally, the two data sources were integrated to construct a comprehensive dataset containing 13,112 images. The dataset includes 1511 images of normal, 324 images of *Bacterial leaf streak*, 293 images of *Bacterial panicle blight*, 1986 images of *Bacterial leaf blight*, 2071 images of *Blast*, 1976 images of *Brown spot*, 1248 images of *Dead heart*, 524 images of *Downy mildew*, 1385 images of *Hispa*, and 1794 images of *Tungro*. As shown in **Figure 2**, the numbers of bacterial leaf streak, bacterial panicle blight, and downy mildew images in the dataset are relatively limited, whereas the distributions of the other categories are comparatively balanced. To address this issue, data augmentation was subsequently applied to these three underrepresented rice disease categories, which helps alleviate the potential bias caused by class imbalance. All data were manually annotated by professionals to ensure the accuracy and reliability of the labeling process. After annotation, all labeled images were systematically stored and organized into category-specific folders for subsequent analysis.

It is worth noting that some rice diseases exhibit substantial similarity in structure and color. For example, *bacterial leaf blight* and *bacterial leaf streak* share similar visual characteristics, while their key differences mainly lie in the lesion location and spot coloration. Therefore, developing a model that can effectively distinguish visually similar rice diseases remains a challenging task.

### 2.2 Data preprocessing

During data preprocessing, all collected images were first resized to 224 × 224 pixels to meet the input requirements of the proposed model. The pixel values were then normalized to accelerate model convergence and improve training stability. To address the insufficient number of samples in some categories, data augmentation strategies, including random rotation, random brightness and contrast perturbation, and random noise injection, were applied to expand the dataset and alleviate the effect of class imbalance. The specific parameter ranges for each of these augmentation operations primarily include a random rotation range of [-90°, 90°], with blank areas filled with zeros, a brightness perturbation coefficient range of [0.6, 1.4] and a contrast perturbation coefficient range of [0.7, 1.3]. Random noise consists of Gaussian noise with a mean of 0 and a standard deviation of 0.02, superimposed onto the normalised image pixels. All enhancement operations are applied with a probability of 0.5 to disease categories with a small sample size, thereby mitigating class imbalance whilst avoiding the generation of distorted images that deviate from real-world field conditions. The revised manuscript will include all the above parameters to improve the details regarding experimental reproducibility.

After augmentation, the dataset was expanded to 17,245 images. Subsequently, the dataset was randomly divided into training, validation, and test sets at a ratio of 6:1:3, respectively. This partitioning strategy was adopted to ensure the rationality and reliability of model training and evaluation. The detailed split results are presented in **Table 1**.

**Table 1:** Dataset split results.

No.	Class	Train	Validation	Test	Total
1	Normal	906	151	454	1511
2	Bacterial leaf streak	1166	194	584	1944
3	Bacterial panicle blight	1054	176	528	1758
4	Bacterial leaf blight	1191	199	596	1986
5	Blast	1242	207	622	2071
6	Brown spot	1185	198	593	1976
7	Dead heart	748	125	375	1248
8	Downy mildew	943	157	472	1572
9	Hispa	831	138	416	1385
10	Tungro	1076	179	539	1794
Total	–	10342	1724	5179	17245

### 2.3 Methodology

**Figure 3:**
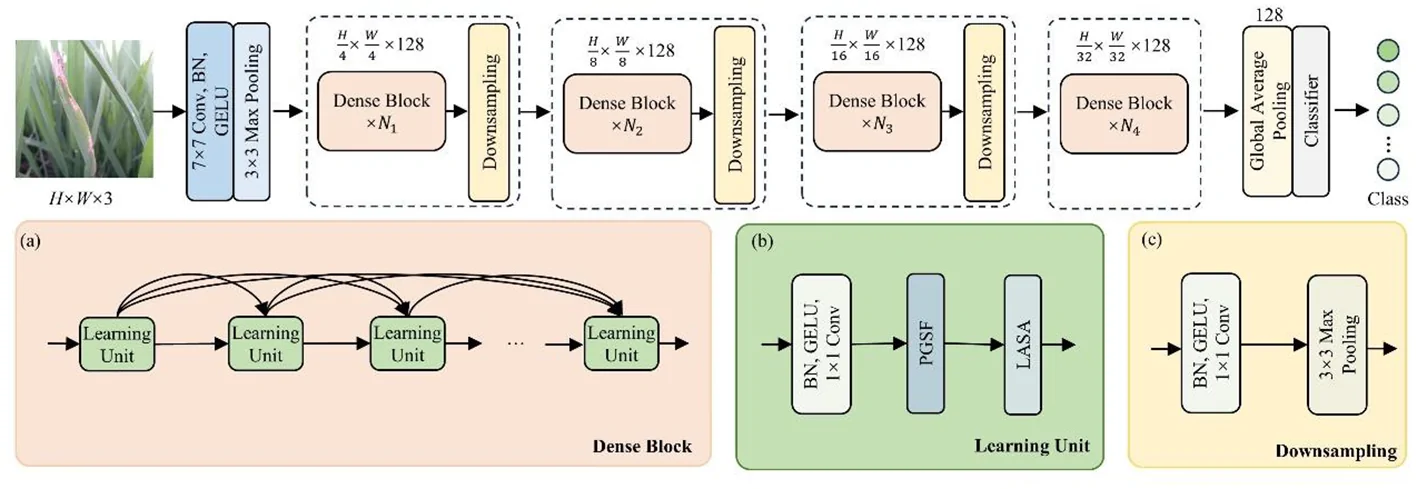
Overall framework of WPMamba. **(a)** Dense block; **(b)** learning unit; **(c)** downsampling.

The proposed WPMamba model for rice disease recognition is illustrated in **Figure 3**. Let the input image be denoted as $X\in {\mathbb{R}}^{H\times W\times 3}$, where *H* and *W* represent the height and width of the image, respectively. First, a convolutional layer is used to extract shallow features from the input image *X*. This layer adopts 64 convolution kernels with a size of 7×7 and a stride of 2, followed by a max-pooling operation with a kernel size of 3×3 and a stride of 2 to downsample the feature maps. In this way, low-level semantic features with relatively high spatial resolution are obtained.

Subsequently, four stages of feature extraction modules are employed to learn deep image representations. Each stage consists of multiple densely connected feature learning units, and downsampling is performed between adjacent stages to reduce the spatial size of feature maps. The downsampling operation includes a 1×1 convolution and a 3×3 max-pooling layer, where the convolution is used to reduce the feature dimensionality and the pooling operation is used to reduce the feature map size. Therefore, after four stages of feature learning, feature maps with four different spatial resolutions are obtained, namely $\frac{H}{4}\times \frac{W}{4}$, $\frac{H}{8}\times \frac{W}{8}$, $\frac{H}{16}\times \frac{W}{16}$, $\frac{H}{32}\times \frac{W}{32}$. Since the preprocessed input images are resized to224×224, the corresponding feature map sizes of the four stages are 56×56,  28×28,  14×14,  7×7 respectively. To reduce the model size, two dense blocks are used in each stage for feature learning. Accordingly, the number of output feature maps in each stage is set to 128.

Each feature learning unit mainly consists of a PGSF module and a LASA module. The PGSF module is designed to adaptively integrate features extracted from different branches, such as frequency-domain and spatial-domain branches. By introducing prior information to guide feature selection, the model can dynamically assign the importance of each branch according to the input, thereby achieving effective feature fusion and complementary representation. The LASA module focuses on feature refinement in the spatial dimension. It enhances the responses of disease-related salient regions while suppressing irrelevant background interference, guiding the network to focus more on lesion areas and improving the discriminative and localization capabilities of learned features.

Similar to the 1×1 convolution used in the downsampling operation, the 1×1 convolution in the feature learning unit is also employed to reduce the feature dimensionality, thereby decreasing computational cost and memory consumption. In addition, batch normalization (BN) is used to stabilize feature distributions and accelerate model convergence, while the GELU activation function enhances feature representation by introducing nonlinear mapping. Finally, global average pooling is applied to obtain the final feature representation, which is then fed into a single fully connected (FC) classifier to distinguish different rice disease categories.

#### 2.3.1 Dense block

In WPMamba, the Dense Block adopts a Dense Connection mechanism to enhance feature reuse and promote efficient information propagation throughout the network. Let the initial input feature of the dense block be denoted as $F_{0}\in {\mathbb{R}}^{H\times W\times C}$, where *C* represents the number of channels. In the dense connection structure, the input of the l-th layer is obtained by concatenating the outputs of all preceding layers along the channel dimension, as formulated below in Equation 1:

(1) $$F_{l}=[F_{0},F_{1},\ldots ,F_{l-1}]$$

where [·] denotes the channel-wise concatenation operation. Therefore, as the number of layers increases, the number of feature channels gradually grows, which facilitates the integration of feature information from different levels. If each feature learning unit outputs *k* channels, the number of feature channels within the dense block after *L* layers can be expressed as Equation 2:

(2) $$C_{L}=C_{0}+L\cdot k$$

This design enables shallow features to directly participate in deep feature representation, effectively alleviating the vanishing gradient problem and improving feature representation capability. In this study, *k* is set to 32; therefore, the number of output feature maps in each stage is 128. In addition, considering the computational overhead caused by the linear growth of channel numbers with increasing layers, a channel compression operation is introduced at the output of each dense block to map the concatenated features into a fixed dimension. This corresponds to the 1×1 convolution shown in **Figure 2b**. In this way, the network can preserve multi-level feature information while effectively controlling model complexity and ensuring consistent output channels across different stages.

#### 2.3.2 The PGSF module

To fully exploit local spatial textures and global frequency-domain priors in rice disease images, this study designs a PGSF module. As shown in **Figure 3**, the proposed module consists of three core components: a local spatial feature extraction branch, a wavelet-prior-guided global branch, and an adaptive selective fusion mechanism.

In the local spatial feature extraction branch, the network aims to capture fine-grained local textures of disease lesions. Given an input feature map $Z\in {\mathbb{R}}^{H\times W\times C}$, local spatial features are extracted using a 3×3 convolutional layer, the BN, and the GELU activation function, which can be formulated as Equation 3:

(3) $$F_{local}=\text{GELU}(\text{BN}({\text{Conv}}_{3\times 3}(Z)))$$

where GELU(·) denotes the GELU activation function, BN(·) denotes the BN layer, and ${\text{Conv}}_{3\times 3}(\cdot )$ represents a convolutional layer with a kernel size of 3×3. The resulting local feature map $F_{local}\in {\mathbb{R}}^{H\times W\times C}$ preserves the original spatial resolution, thereby retaining precise disease edge and location information.

In the wavelet-prior-guided global branch, the module introduces the two-dimensional discrete wavelet transform (DWT) as a frequency-domain prior to guide the Mamba model in capturing global long-range dependencies more efficiently. First, DWT decomposes the input *Z* into four sub-bands containing different frequency information, namely the low-frequency approximation component $I_{ll}$ and the high-frequency detail components $I_{hl}$, $I_{lh}$, $I_{hh}$. This process can be expressed as Equation 4:

(4) $$\left\{I_{ll},I_{hl},I_{lh},I_{hh}\}=\text{DWT}(Z\right)$$

where DWT(·) denotes the two-dimensional discrete wavelet transform. Subsequently, the four sub-bands are concatenated along the channel dimension to obtain the fused frequency-domain feature $Z_{freq}\in {\mathbb{R}}^{\frac{H}{2}\times \frac{W}{2}\times 4C}$.

To establish a global receptive field, $Z_{freq}$ is reshaped into a one-dimensional sequence and then fed into the Mamba state space model. Owing to its linear-complexity modeling capability, Mamba can efficiently integrate frequency-domain features across distant regions. The detailed principles of Mamba can be found in the study by Gu and Dao, 2024. After the output sequence is inversely reshaped, a pixel shuffle operation is applied to remap channel-wise information into the spatial dimension, thereby restoring the spatial resolution of the feature map without information loss. This process can be formulated as Equation 5:

(5) $$F_{global}=\text{PixelShuffle}(\text{Reshape}(\text{Mamba}(\text{Reshape}(Z_{freq}))))$$

where PixelShuffle(·) denotes the pixel shuffle operation, Reshape(·) represents dimensional reshaping, and Mamba(·) denotes the Mamba encoding function.

**Figure 4:**
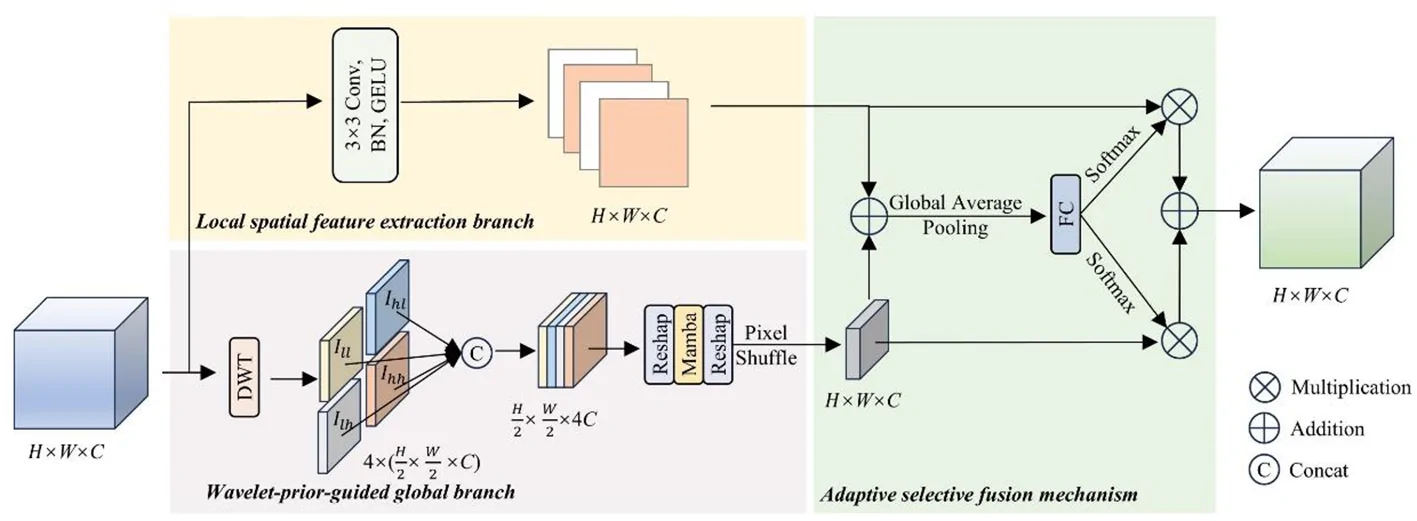
Structure of the PGSF module.

Next, to enable the network to adaptively select the most informative feature domain according to disease characteristics, the PGSF module adopts a selective fusion strategy based on dynamic channel attention, as illustrated in the adaptive selective fusion mechanism in **Figure 4**. First, the features from the two branches are integrated through element-wise addition to combine spatial-frequency information. Then, global average pooling (GAP) is used to extract channel-wise statistical information $S\in {\mathbb{R}}^{C}$, which can be formulated as Equation 6:

(6) $$S=\text{GAP}(F_{local}⊕F_{global})$$

where GAP(·) denotes global average pooling, and ⊕ represents element-wise addition. Subsequently, the statistical descriptor *S* is fed into an FC layer and normalized along the branch dimension using the Softmax function, generating dynamic channel-wise weight vectors for the local and global features, denoted as $w_{local},w_{global}\in {\mathbb{R}}^{C}$, as Equation 7:

(7) $$\left[w_{local},w_{global}]=\text{Softmax}(\text{FC}(S)\right)$$

where Softmax(·) denotes the Softmax function, and FC(·) represents the fully connected layer. It is worth noting that, for the *c*-th channel, the generated weights satisfy $w_{local}^{\left(c\right)}+w_{global}^{\left(c\right)}=1$. Finally, the generated weights are used to modulate the original features through element-wise multiplication, and the final fused output *Y* is obtained by summation, formulated as Equation 8:

(8) $$Y=(w_{local}⊗F_{local})⊕(w_{global}⊗F_{global})$$

where ⊗ denotes element-wise multiplication. Through this process, the wavelet prior effectively guides feature flow, enabling the network to rely more on global frequency-domain features when processing smooth regions with complex backgrounds, while focusing more on local spatial textures around lesion boundaries. This improves the model’s ability to recognize complex rice diseases.

#### 2.3.3 The LASA module

**Figure 5:**
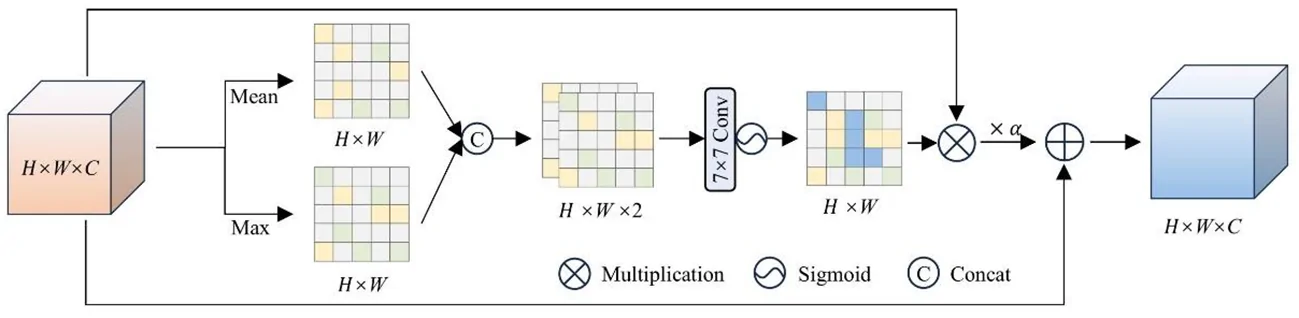
Structure of the LASA module.

In complex rice field backgrounds, the LASA module is designed to enable the network to accurately focus on subtle lesion regions while suppressing irrelevant noise. As shown in **Figure 5**, this module aggregates channel information along the spatial dimension and introduces learnable dynamic parameters for residual enhancement, thereby achieving fine-grained localization and refinement of disease-related features.

Given the input feature $Y\in {\mathbb{R}}^{H\times W\times C}$, average pooling and max pooling are first performed along the channel dimension to obtain two spatial descriptors, as Equations 9, 10:

(9) $$F_{\text{avg}}=\text{AvgPool}(Y)$$

(10) $$F_{\text{max}}=\text{MaxPool}(Y)$$

where AvgPool(·) denotes average pooling, and MaxPool(·) denotes max pooling. In this process, max pooling helps highlight high-frequency lesion boundaries, whereas average pooling provides smooth contextual information from the background and lesion interiors. Both $F_{\text{avg}}$ and $F_{\text{max}}$ are two-dimensional spatial feature maps that characterize feature responses from different perspectives. The two descriptors are then concatenated along the channel dimension, as Equation 11:

(11) $$F_{c}=[F_{\text{avg}},F_{\text{max}}]$$

where [·] denotes the channel-wise concatenation operation. To effectively exploit neighborhood information from surrounding pixels, a 7×7 convolutional layer with a large receptive field is applied to the concatenated descriptors for spatial mapping. Then, a Sigmoid activation function is used to normalize the values and generate the final two-dimensional spatial attention mask $A\in {\mathbb{R}}^{H\times W}$, as formulated below in Equation 12:

(12) $$A=\sigma (\text{C}on{\text{v}}_{7\times 7}(F_{c}))$$

where σ(·) denotes the Sigmoid activation function, $\text{C}on{\text{v}}_{7\times 7}(\cdot )$ represents a convolutional layer with a kernel size of 7×7, and *A* denotes the spatial attention map. Finally, the attention weights are applied to the input feature to enhance key regions, as Equation 13:

(13) $$F^{\prime }=A⊗Y$$

To prevent the attention mechanism from disrupting the integrity of the original features during the early training stage, a learnable scalar parameter *α* is introduced, and a residual structure is adopted for final feature fusion, expressed as Equation 14:

(14) $$F_{\text{out}}=Y+\alpha F^{\prime }$$

Through this process, the LASA module can adaptively highlight the responses of lesion regions and enhance the model’s perception of fine-grained disease features, thereby improving the overall recognition performance.

### 2.4 Experimental settings and evaluation metrics

All experiments were implemented using Python 3.10 and the PyTorch 2.1.2 deep learning framework. The experiments were conducted on a GPU platform equipped with an NVIDIA RTX 3090 GPU with 24 GB of memory. An Intel Xeon Platinum 8358P CPU operating at 2.60 GHz and 90 GB of RAM were also used to ensure efficient and stable model training.

During training, the Adam optimizer was adopted for parameter updating. The initial learning rate was set to 0.001, the number of training epochs was set to 100, and the batch size was set to 32. The cross-entropy loss function was used as the optimization objective. In addition, to improve the reliability and generalization of the experimental results, five-fold cross-validation was employed for model evaluation.

Accuracy, Precision, Recall, and F1-score were selected as evaluation metrics. Accuracy measures the overall classification correctness, whereas Precision, Recall, and F1-score reflect classification performance from different perspectives across categories. These metrics provide a more comprehensive evaluation of model performance in the rice disease recognition task. They are calculated as follows in Equations 15–18:

(15) $$\text{Accuracy}=\frac{TP+TN}{TP+TN+FP+FN}$$

(16) $$\text{Precision}=\frac{TP}{TP+FP}$$

(17) $$\text{Recall}=\frac{TP}{TP+FN}$$

(18) $$\text{F}1-\text{Score}=\frac{2\times \text{Precision}\times \text{Recall}}{\text{Precision}+\text{Recall}}$$

where TP (true positive) denotes the number of samples correctly predicted as positive, TN (true negative) denotes the number of samples correctly predicted as negative, FP (false positive) denotes the number of samples incorrectly predicted as positive, and FN (false negative) denotes the number of samples incorrectly predicted as negative.

## 3 Results

### 3.1 Ablation study

To further analyze the effectiveness of each component in WPMamba, ablation experiments were conducted, and the results are presented in **Table 2**. As shown in the table, each module contributes to a noticeable improvement in model performance, with an overall gradually increasing trend.

**Table 2:** Results of the ablation experiments.

CNN branch	Mamba branch	Branch fusion	LASA	Precision (%)	Recall (%)	F1-Score (%)	Accuracy (%)
✓				93.58	93.76	93.64	93.76
	✓			94.15	94.30	94.20	94.32
✓	✓			94.62	94.85	94.73	94.83
✓	✓	✓		95.01	95.22	95.09	95.17
✓	✓	✓	✓	95.60	95.77	95.67	95.72

First, when only the CNN branch is used, the model already shows a certain feature extraction capability, achieving an Accuracy of 93.76%. This indicates that spatial-domain features provide a fundamental basis for rice disease recognition. When only the Mamba branch is used, the performance is slightly better than that of the CNN branch, with an Accuracy of 94.32%, suggesting that features based on frequency-domain information and global modeling are more advantageous in capturing the overall structural information of disease symptoms. When the CNN and Mamba branches are introduced simultaneously, the model Accuracy further increases to 94.83%, demonstrating that spatial-domain and frequency-domain features are complementary and can enhance feature representation from different perspectives.

On this basis, after adding the branch fusion module, the Accuracy improves to 95.17%. This shows that the designed selective fusion mechanism can effectively integrate the two types of features, avoid information redundancy caused by simple feature addition, and achieve better feature collaboration. Finally, after introducing the LASA module, the model achieves the best performance, with an Accuracy of 95.72% and an F1-score of 95.67%. This indicates that the LASA module can further highlight key disease regions and suppress background interference, thereby improving the model’s discriminative ability for fine-grained features.

Overall, each component plays a positive role in improving model performance. In particular, the branch fusion and LASA modules contribute prominently to accuracy improvement, validating the effectiveness of the proposed method in feature modeling and key region perception.

**Table 3** further analyzes the influence of different pooling strategies in the downsampling operation on model performance. When average pooling is used, the overall performance of the model is relatively lower, with an Accuracy of 94.67% and an F1-score of 94.57%. In contrast, when max pooling is adopted, all evaluation metrics improve noticeably, with the Accuracy reaching 95.72% and the F1-score increasing to 95.67%. This indicates that max pooling is more effective in preserving discriminative features for the current task.

**Table 3:** Influence of different pooling strategies in downsampling on model performance.

Method	Precision (%)	Recall (%)	F1-Score (%)	Accuracy (%)
WPMamba with average pooling	94.56	94.66	94.57	94.67
WPMamba with max pooling	95.60	95.77	95.67	95.72

The reason is that average pooling smooths local regions during downsampling, which may weaken highly responsive and discriminative disease-related features, such as lesion edges and texture variations. By comparison, max pooling retains the strongest response within each local region, helping to highlight key lesion information. Therefore, in rice disease recognition, which is sensitive to fine-grained features, max pooling can more effectively enhance important feature representations and improve overall model performance. This also verifies the rationality of adopting max pooling as the downsampling strategy in the WPMamba architecture.

### 3.2 Comparison with other methods

To verify the effectiveness of WPMamba, several mainstream models were selected for comparative experiments, covering CNNs, lightweight networks, Transformer-based models, and Mamba-based models. The CNN models include ResNet101 (He et al., 2015) and EfficientNet (Tan and Le, 2019). The lightweight networks include MobileNetV3 (Howard et al., 2019), ShuffleNet (Zhang et al., 2018), and GhostNet (Han et al., 2020). The Transformer-based models include DeiT-tiny (Touvron et al., 2021), MobileViT (Mehta and Rastegari, 2022), and RepViT (Wang et al., 2024). The Mamba-based models include ResMamba (Zhang et al., 2025c) and ViM (Zhu et al., 2024). The parameters of all comparison models were set according to their original papers.

As shown in **Table 4**, the proposed WPMamba achieves the best performance across all evaluation metrics, with an Accuracy of 95.72% and an F1-score of 95.67%, clearly outperforming the other comparison methods. Compared with conventional CNN models, such as EfficientNet and ResNet101, WPMamba maintains strong local feature extraction capability while further improving feature representation by introducing wavelet priors and a global modeling mechanism. Compared with the second-best model, EfficientNet, WPMamba improves Accuracy and F1-score by 1.57% and 1.58%, respectively. Compared with lightweight networks, such as MobileNetV3 and ShuffleNet, the proposed method shows a more obvious advantage in recognition accuracy, indicating its stronger robustness in modeling complex disease features.

**Table 4:** Results of different comparison methods.

Method	Precision (%)	Recall (%)	F1-Score (%)	Accuracy (%)
DeiT-tiny	90.79	90.77	90.74	90.98
EfficientNet	94.09	94.18	94.09	94.15
GhostNet	92.71	92.95	92.76	92.88
Mobilenetv3	94.02	94.01	93.98	94.11
MobileViT	91.26	91.09	91.12	91.25
RepViT	87.23	87.51	87.20	87.35
ResMamba	74.07	73.75	73.56	74.78
ResNet101	92.59	92.88	92.67	92.78
ShuffleNet	91.93	91.92	91.87	92.03
ViM	76.48	76.55	76.48	77.25
WPMamba	95.60	95.77	95.67	95.72

For Transformer- and Mamba-based models, such as DeiT-tiny, MobileViT, ResMamba, and ViM, their relatively lower performance suggests that relying solely on global modeling or sequence modeling is insufficient to fully capture fine-grained disease texture information in this task. Overall, by integrating spatial-domain and frequency-domain features and combining prior guidance with an attention mechanism, WPMamba achieves more precise modeling of key rice disease characteristics. Consequently, it outperforms existing methods across all metrics, validating the effectiveness and superiority of the proposed model.

**Figure 6:**
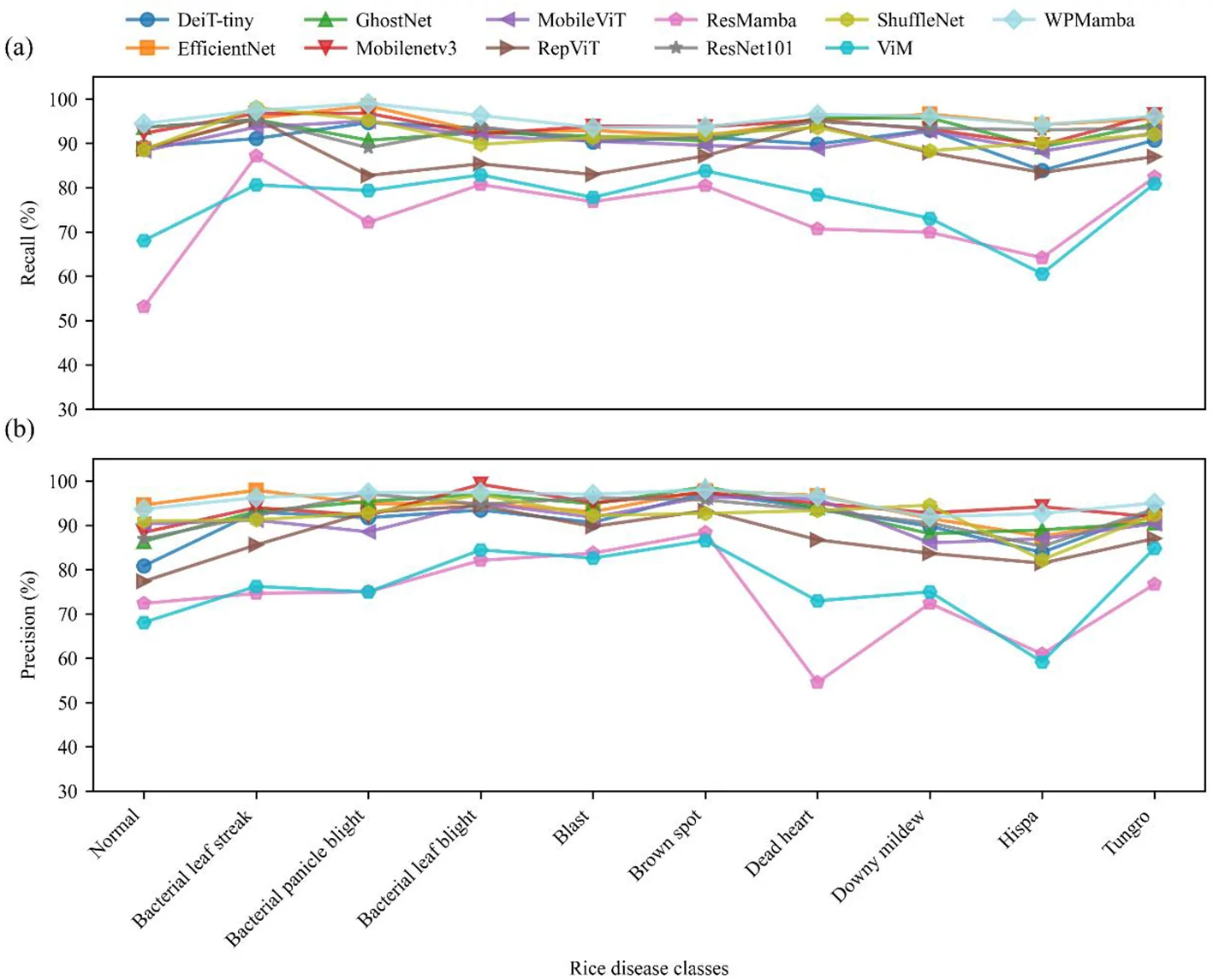
Precision and recall results for each category. **(a)** Precision. **(b)** Recall.

**Figure 6** further analyzes the Precision and Recall of each category. As shown in the figure, different models exhibit certain performance variations across rice disease categories, while the overall trend remains relatively consistent. Among them, WPMamba maintains high and stable Precision and Recall values for most categories, demonstrating superior overall performance.

For typical disease categories such as bacterial leaf streak, blast, and brown spot, WPMamba achieves near-optimal or optimal values for both metrics, indicating its ability to effectively capture key disease characteristics. For relatively complex or easily confused categories, such as hispa and dead heart, some comparison models show obvious fluctuations or even performance degradation. In contrast, WPMamba still maintains high recognition precision and recall, reflecting stronger robustness and generalization capability.

By comparison, some models, such as ResMamba and ViM, show evident performance fluctuations across multiple categories. In particular, their Precision or Recall drops substantially for categories such as dead heart and hispa, suggesting insufficient capability in modeling fine-grained textures or local lesion regions. Conventional CNN models, such as EfficientNet and ResNet101, show relatively stable overall performance, but they remain slightly inferior to WPMamba in some categories, indicating certain limitations in global information modeling or multi-scale feature fusion.

Overall, WPMamba achieves a better balance and stability between Precision and Recall across different categories. It can more effectively extract discriminative features from different types of rice diseases, thereby achieving superior performance in multi-class recognition tasks.

**Figure 7:**
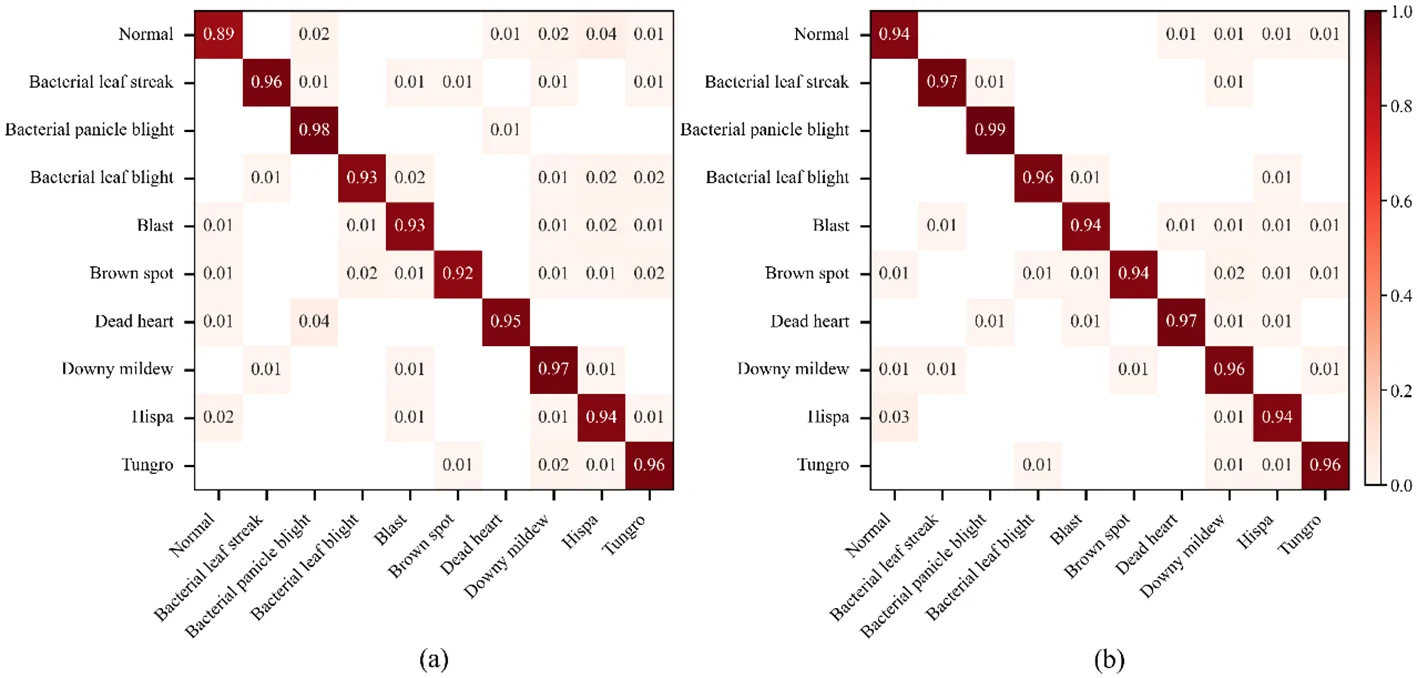
Comparative analysis of confusion matrices. **(a)** EfficientNet. **(b)** WPMamba.

To intuitively visualize inter-class misclassification, we selected EfficientNet, the model achieving the highest recognition accuracy among all comparison methods, and plotted the corresponding confusion matrices, as shown in **Figure 7**. Confusion matrix results demonstrate that both EfficientNet and WPMamba attain high accuracy for rice disease identification, with classification accuracy exceeding 90% for most categories. Compared with EfficientNet, WPMamba exhibits stronger inter-class discriminative ability, manifested by more concentrated values along the diagonal, which indicates that the proposed model can accurately distinguish various disease types. Notably, the recognition accuracies of WPMamba for easily confused categories, including Normal, Bacterial leaf blight and Brown spot, reach 0.94, 0.96 and 0.94, respectively. This effectively alleviates misclassification between healthy leaves and diseased leaves, as well as among similar disease types.

**Figure 8:**
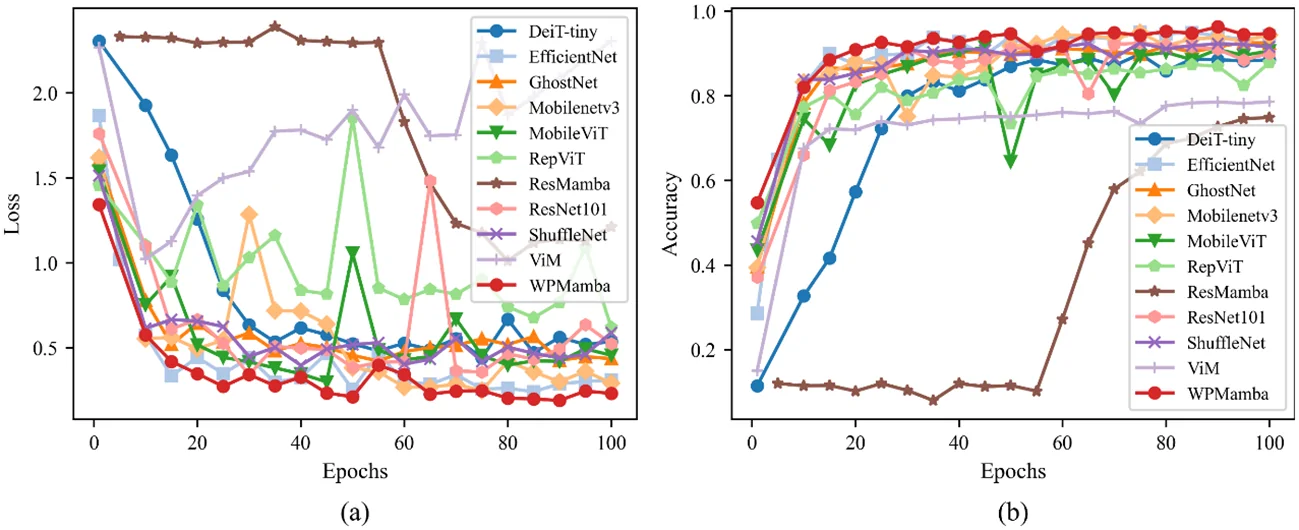
Loss and accuracy curves of different models on the test set. **(a)** Loss. **(b)** Accuracy.

As shown by the loss and accuracy curves on the test set in **Figure 8**, different models exhibit clear differences in convergence speed and stability. Overall, WPMamba shows a rapid decrease in loss during the early training stage and becomes stable at an earlier epoch, with the final loss remaining at a relatively low level. This indicates that the proposed model has favorable convergence behavior. Meanwhile, its accuracy curve continues to increase and stabilizes at a high level, eventually achieving the best performance, which demonstrates good generalization capability and stability.

By contrast, some models, such as DeiT-tiny, ResMamba, and ViM, converge more slowly or show larger fluctuations during training. For example, ResMamba maintains a relatively low accuracy in the early stage and improves noticeably only in the later stage, indicating poorer training stability. ViM exhibits obvious fluctuations in the middle and late training stages, suggesting that it is more sensitive to changes in data distribution. Conventional CNN models, such as EfficientNet and ResNet101, generally converge relatively quickly, but still show certain fluctuations in the later stage, making them slightly less stable than WPMamba.

Overall, the proposed WPMamba not only achieves faster convergence but also maintains lower loss and higher, more stable accuracy during training. These results indicate its advantages in feature modeling and optimization.

### 3.3 Feature visualization

**Figure 9:**
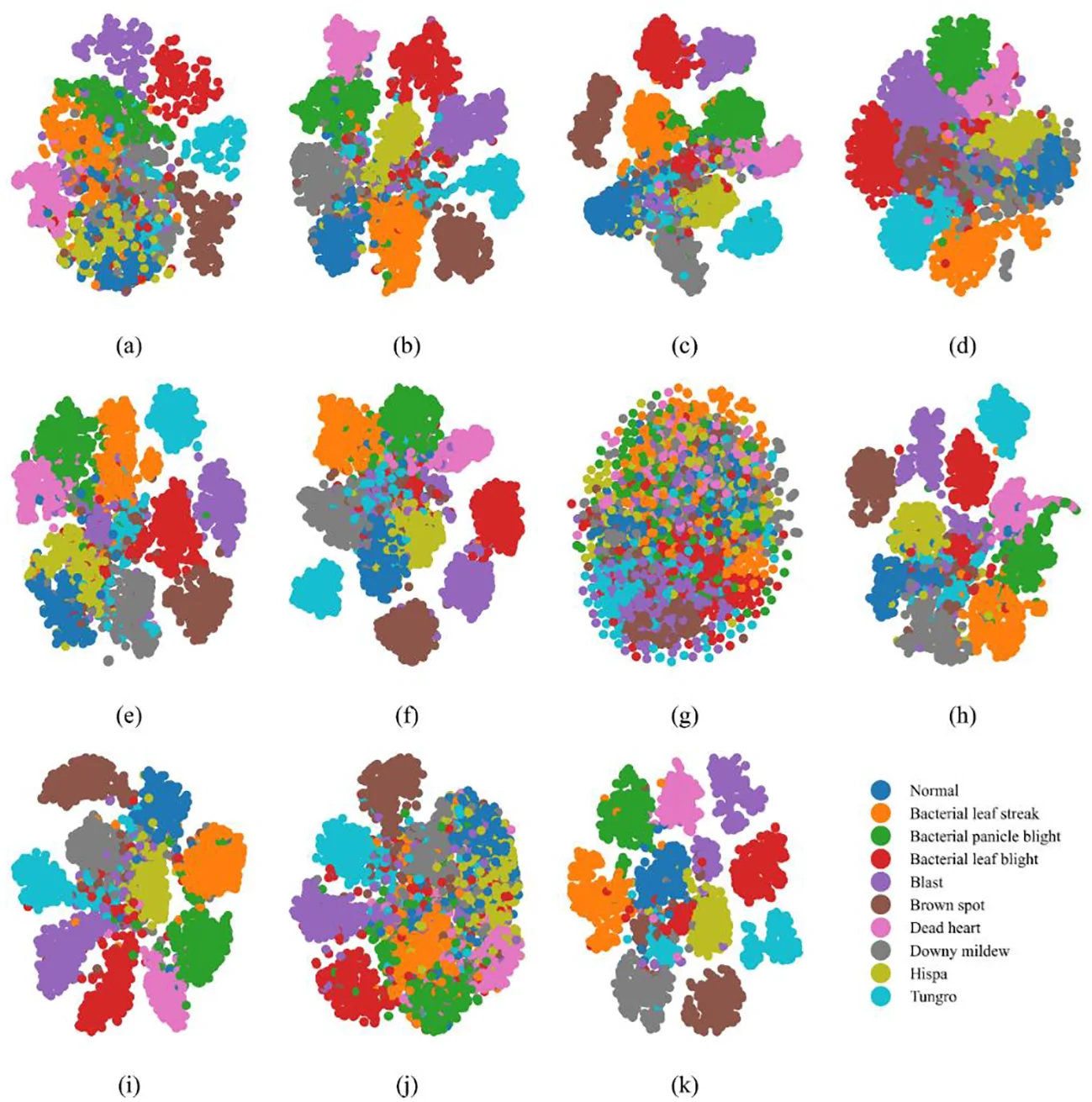
t-SNE visualization of the features extracted by different models. **(a)** DeiT-tiny. **(b)** EfficientNet. **(c)** GhostNet. **(d)** Mobilenetv3. **(e)** MobileViT. **(f)** RepViT. **(g)** ResMamba. **(h)** ResNet101. **(i)** ShuffleNet. **(j)** ViM. **(k)** WPMamba.

To further analyze the recognition capability of the models for rice diseases, t-SNE was used to visualize the extracted features. As shown in **Figure 9**, the proposed WPMamba (**Figure 9k**) exhibits the clearest category clustering structure in the feature space. Most samples from different categories form compact and well-separated clusters, indicating that the learned features have stronger discriminability and consistency. This suggests that WPMamba can effectively distinguish different types of rice diseases and reduce inter-class confusion.

In contrast, the other models generally show feature overlap to varying degrees. For example, in DeiT-tiny (**Figure 9a**), MobileViT (**Figure 9e**), and ViM (**Figure 9j**), different categories are heavily mixed, indicating insufficient feature representation capability. ResMamba (**Figure 9g**) even shows a highly aggregated distribution without clear class boundaries, suggesting that it fails to learn sufficiently discriminative features, which is consistent with the loss results shown in **Figure 8**. Conventional CNN models such as EfficientNet (**Figure 9b**) and ResNet101 (**Figure 9h**) can form category clusters to some extent, but local overlaps are still observed, and the inter-class separation remains insufficient. Lightweight models such as GhostNet (**Figure 9c**), ShuffleNet (**Figure 9i**), and MobileNetV3 (**Figure 9d**) show favorable clustering trends in some categories, but their overall feature distributions remain relatively scattered.

By effectively integrating spatial-domain and frequency-domain information and introducing prior guidance and attention mechanisms, the proposed WPMamba is able to learn more discriminative feature representations.

### 3.4 Visualization of recognition results

**Figure 10:**
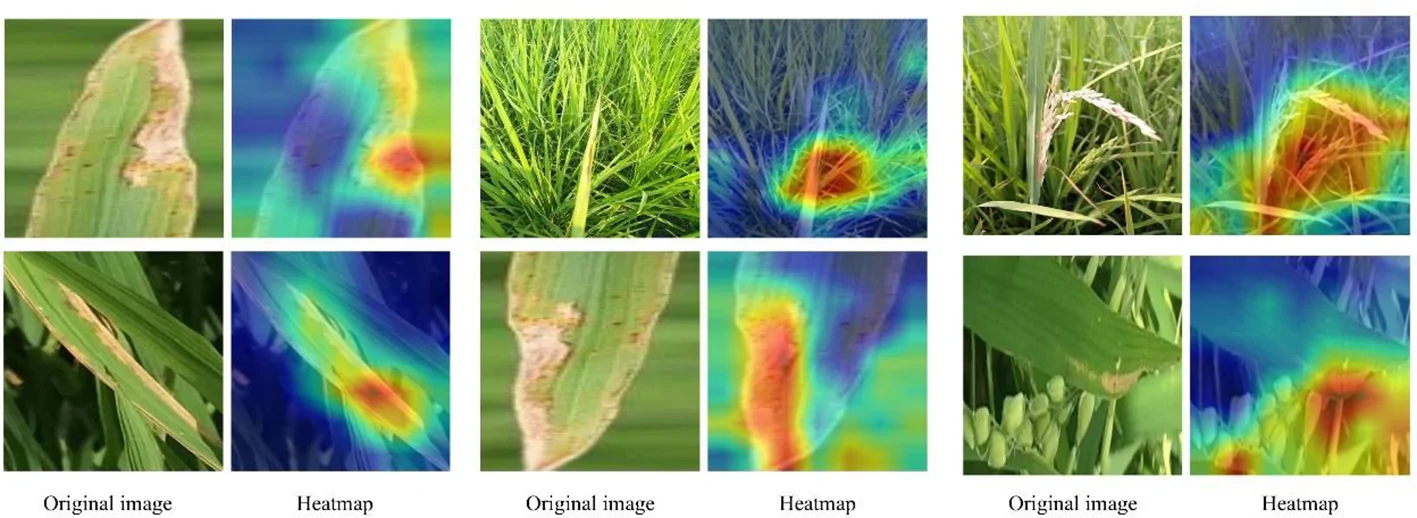
Heatmap visualization of the recognition results.

The study further uses the Grad-CAM method to visualize the recognition results of rice diseases through heatmaps. As shown in **Figure 10**, WPMamba can accurately focus on the key regions of rice diseases, demonstrating strong discriminative capability. The high-response areas in the heatmaps are mainly concentrated on lesion locations and their surrounding regions on the leaves, such as spots, withered or yellowed areas, and the edges of disease spread, while showing weak responses to non-diseased regions. This indicates that the model can effectively utilize disease-relevant salient features for decision-making rather than relying on irrelevant information.

Further observation shows that the model can adaptively adjust its attention regions for different types of disease images. For example, in images with obvious spot or streak characteristics, the heatmaps closely align with the lesion contours. In cases where the disease distribution is more diffuse, the model can cover a larger range of relevant regions, reflecting its ability to perceive multi-scale disease characteristics. This stable and accurate attention distribution demonstrates that WPMamba can effectively combine spatial information with frequency-domain priors during feature extraction, thereby enhancing its ability to capture fine-grained texture and structural variations.

### 3.5 Computational complexity

To further analyze the computational complexity of the models, FLOPs, Parameters, and Test time were selected for comparison, as shown in **Table 5**. The results indicate that WPMamba achieves a favorable balance between performance and efficiency.

**Table 5:** Computational complexity of different models.

Model	FLOPs (G)	Parameters (M)	Test time (s)	Accuracy (%)
DeiT-tiny	0.92	5.53	32.08	90.98
EfficientNet	2.89	20.19	28.88	94.15
GhostNet	0.15	3.91	23.23	92.88
Mobilenetv3	0.23	4.21	133.89	94.11
MobileViT	1.81	5.00	27.71	91.25
RepViT	4.60	22.41	16.29	87.35
ResMamba	1.61	13.76	65.99	74.78
ResNet101	7.86	42.52	31.43	92.78
ShuffleNet	0.30	2.49	21.28	92.03
ViM	0.06	25.43	18.46	77.25
WPMamba	0.49	1.21	25.51	95.72

First, in terms of model size, WPMamba contains only 1.21 M parameters, which is significantly lower than most comparison models, such as ResNet101 with 42.52 M parameters and EfficientNet with 20.19 M parameters. This demonstrates that the proposed architecture is more lightweight. Meanwhile, its computational cost is 0.49 G FLOPs. Although this is slightly higher than that of some extremely lightweight models, such as GhostNet and ShuffleNet, it still remains at a relatively low level, indicating that the computational overhead is well controlled.

In terms of test time, WPMamba requires 25.51 s, which places it at a moderately favorable level overall and makes it clearly more efficient than some computationally complex or structurally less efficient models, such as MobileNetV3 and ResMamba. This suggests good potential for practical application. It is worth noting that although some models, such as ViM and GhostNet, have lower FLOPs or fewer parameters, their classification accuracy is obviously inferior, indicating that simply reducing model complexity often leads to performance degradation.

Overall, WPMamba achieves the highest classification accuracy of 95.72% while maintaining a low parameter count and moderate computational complexity. Compared with other models, it offers a more advantageous trade-off between accuracy and complexity.

All models were inferred uniformly on a single NVIDIA RTX 3090 (24 GB) GPU via PyTorch 2.1.2, with mixed precision and quantization disabled for fair comparison; the CPU only handled data scheduling without network computation. Inference batch size was fixed at 32, matching the training setup. The listed test time represents the total pure forward latency for all 5179 test images, excluding offline preprocessing steps (resizing, normalization) that were pre-computed and cached. All comparison models used official implementations with a unified 224×224 input size, without extra augmentation or network pruning to avoid confounding variables.

## 4 Discussions

The proposed WPMamba achieves a favorable balance between feature representation capability and model efficiency through its architectural design, thereby obtaining strong performance with relatively low complexity. Specifically, the PGSF module employs a dual-branch structure in the spatial and frequency domains to jointly model local details and global information. The convolution-based spatial branch effectively extracts local characteristics of disease regions, such as lesion edges and textures, whereas the wavelet-based frequency branch decomposes input features into different frequency components, thereby introducing physically meaningful prior information. The low-frequency components help characterize the overall leaf structure, while the high-frequency components emphasize lesion details. Compared with conventional convolution, this multi-scale decomposition strategy is more suitable for handling the complex and fine-grained texture variations in rice disease images. In addition, Mamba is introduced to model the frequency-domain features, allowing global dependencies to be established across different frequency components. This compensates for the limited long-range modeling capability of convolution and further enhances the consistency and completeness of feature representation.

Considering the differences between the two branches in feature learning, a selective adaptive mechanism is adopted to dynamically weight spatial-domain and frequency-domain information rather than simply combining them. This strategy can adaptively adjust the contributions of the two types of features according to the feature distribution of different input samples, thereby avoiding interference from redundant information and achieving more efficient feature integration. Meanwhile, the LASA module further refines feature representation in the spatial dimension by explicitly modeling the importance of lesion regions, guiding the model to focus on key areas while suppressing background noise. In other words, after the disease image features are learned, the LASA module performs regional filtering on the fused feature maps so that the final features become more discriminative. Moreover, WPMamba adopts a stage-wise architecture and uses dense connections for feature reuse, ensuring sufficient information flow while avoiding the redundant computation caused by excessively deep networks. This design enables the model to learn rich and effective feature representations with a relatively low number of parameters and low computational complexity. Combined with the experimental results presented above, it can be seen that the collaborative effects of the proposed modules in feature modeling, fusion, and selection are the main reasons for the performance improvement. Overall, by introducing wavelet priors, a global modeling mechanism, and adaptive fusion and attention strategies, WPMamba achieves stronger rice disease recognition capability while maintaining high efficiency, providing an effective solution for intelligent diagnosis in complex agricultural scenarios.

Although WPMamba achieves promising performance in rice disease recognition, it still has some limitations. On the one hand, although the LASA module can enhance the model’s attention to lesion regions, attention drift may still occur when disease characteristics are inconspicuous or background interference is strong. To address this limitation, future work may incorporate more refined region-constrained mechanisms, such as weakly supervised localization information or multi-scale attention mechanisms, to further improve the model’s adaptability to complex scenarios. On the other hand, the dataset used in the current study remains relatively limited. Although a cross-validation strategy was adopted, the generalization capability of the model under cross-region and cross-environment conditions still requires further verification. Future work could evaluate the robustness of the proposed model under different environmental conditions by constructing multi-source datasets or conducting cross-dataset experiments. Nevertheless, the proposed WPMamba still provides a useful reference for rice disease recognition.

## 5 Conclusions

This study addresses the difficulty of effectively modeling fine-grained features in rice disease recognition and proposes a Wavelet-Prior-Guided Mamba network, termed WPMamba. The proposed method introduces wavelet decomposition to obtain frequency-domain prior information and combines it with Mamba for global feature modeling. In addition, a prior-guided selective fusion (PGSF) module is designed to achieve adaptive fusion of spatial-domain and frequency-domain features, while a lesion-aware spatial attention (LASA) module is employed to enhance the model’s focus on lesion regions, thereby improving the discriminative capability of feature representations.

Experimental results demonstrate that WPMamba outperforms several mainstream methods across multiple evaluation metrics. It achieves higher recognition accuracy while maintaining a low parameter count and low computational complexity. Furthermore, ablation experiments validate the effectiveness of each proposed module. Overall, the proposed method shows strong performance and promising application potential in rice disease recognition and can provide effective support for intelligent agricultural diagnosis.

## Data Availability

The datasets presented in this study can be found in online repositories. The names of the repository/repositories and accession number(s) can be found below: https://data.mendeley.com/datasets/fwcj7stb8r/1.
